# Patient-Centered Activities Performed by Clinical Research Nurses in Clinical Trials: A Scoping Review of Enacted Practice

**DOI:** 10.3390/healthcare14183111

**Published:** 2026-09-20

**Authors:** Catia Genna, Kiara Ros Thekkan, Silvia Rodelli, Valeria Amatucci, Laura Turchini, Luciana Nicola Giordano, Mattia Bozzetti, Daniele Napolitano

**Affiliations:** 1 Professional Development, Continuing Education and Research Unit, Medical Directorate, Bambino Gesù Children’s Hospital IRCCS, 00165 Rome, Italy; 2 Division of Gastroenterology, S. Camillo Hospital, 00152 Rome, Italy; 3 U.O.C. Oculistica, Fondazione Policlinico Universitario Agostino Gemelli IRCCS, 00168 Rome, Italy; silvia.rodelli@policlinicogemelli.it; 4 CEMAD-Fondazione Policlinico Gemelli IRCCS, 00168 Rome, Italy; valeria.amatucci@policlinicogemelli.it (V.A.); laura.turchini@policlinicogemelli.it (L.T.); luciananicola.giordano@policlinicogemelli.it (L.N.G.); 5 Direction of Health Professions, ASST Cremona, 26100 Cremona, Italy; mattia.bozzetti@asst-cremona.it; 6 SITRA—Scientific Direction, Fondazione Policlinico Gemelli IRCCS, 00168 Rome, Italy; daniele.napolitano@policlinicogemelli.it

**Keywords:** clinical research nursing, patient-centered activities, clinical trials, competencies, professional identity

## Abstract

**Highlights:**

**What are the main findings?**

- This review mapped how patient-centered Clinical Research Nurse competencies are enacted and reported across clinical trial settings.
- CRNs were consistently described as supporting participant care, safety, education, and follow-up, while their level of autonomy varied across studies and healthcare contexts.

**What are the implications of the main findings?**

- The review describes how patient-centered CRN activities are enacted in clinical trial practice, providing an empirical perspective beyond competency frameworks.
- The findings may support future evaluation of how Clinical Research Nurse activities are implemented across different clinical trial contexts and inform education and role development to strengthen patient-centered CRN practice.

**Abstract:**

**Background/Objective:** Clinical Research Nurses (CRNs) are recognized members of multidisciplinary teams in clinical trials (CTs), contributing to both research procedures and patient-centered care. Although competency frameworks describe the expected domains of CRN practice, less is known about how these competencies are enacted in patient-centered activities during CTs. This scoping review aimed to examine how patient-centered activities are enacted by Clinical Research Nurses in clinical trials. **Methods:** A scoping review was conducted following the Joanna Briggs Institute methodology and guided by the PRISMA-ScR checklist. A literature search was performed in MEDLINE, CINAHL, and EMBASE, complemented by gray literature searches across Google Scholar, GreyLit, and clinical research nursing association websites. Studies published in English over the last 20 years were included if they described patient-centered activities enacted by CRNs within CTs. **Results:** Twenty-one studies were included, reporting data from more than 1800 participants, primarily from survey-based research conducted across North and South America, Europe, Asia, Australia and New Zealand. CRNs were frequently described as involved in direct patient care, safety monitoring, patient education, follow-up coordination, and investigational product management. Variability across studies was reported in the implementation and level of autonomy associated with these activities. Studies from the UK, USA, Australia, Canada, Sweden, and New Zealand described CRNs as independently involved in activities such as informed consent and recruitment, whereas studies from Italy and South Korea more frequently reported physician-supported or supervised involvement in these tasks. **Conclusions:** This scoping review mapped how patient-centered activities performed by CRNs are described across clinical trial contexts. The findings highlight the broad range of relational, clinical, educational, and coordinative activities attributed to CRNs in supporting research participants throughout clinical trials, while also identifying variation in how these activities are enacted across studies and settings. The review contributes to the current understanding of the patient-centered dimension of CRN practice and supports further research exploring how these activities are implemented across different clinical trial contexts.

## 1. Introduction

Clinical research constitutes the fundamental basis of medical progress, enabling the assessment of the efficacy and safety of drugs and medical devices [1,2,3,4]. The increasing complexity of clinical trials (CTs) has heightened the need for higher-quality standards in their design, management, and conduct [5]. This has led to the establishment of highly qualified multidisciplinary teams involved throughout the trial process. In this context, Clinical Research Nurses (CRNs) play a pivotal role [6,7].

Over the past two decades, the roles and responsibilities of CRNs have been more clearly defined and documented in the literature, highlighting their complex and multidimensional scope of practice [8]. The National Institutes of Health Clinical Center defined the CRN domain of practice, detailing competencies across five dimensions: (i) clinical practice, (ii) care coordination and continuity, (iii) human subjects protection, (iv) study management, and (v) contribution to science [9,10]. In 2016, the American Nurses Association (ANA) and the International Association of Clinical Research Nurses (IACRN) officially recognized CRN practice as a nursing specialty, highlighting its critical role in patient safety and protocol adherence [11,12]. The CRN bridges clinical practice and research, balancing direct patient care with protocol fidelity to safeguard the quality and integrity of CTs [3,7,13].

Beyond protocol implementation, CRNs contribute substantially to the patient-centered dimension of clinical research. Patient-centered care has been recognized as a core component of healthcare quality and refers to care that is respectful of, and responsive to, individuals’ preferences, needs, and values. It also emphasizes the active participation of individuals in clinical decision-making [14]. Within clinical trials, this perspective becomes particularly relevant as participants navigate complex information, demanding procedures, uncertainty regarding outcomes, and ethical considerations associated with research participation. In this context, CRNs frequently act as educators, advocates, coordinators, and trusted points of contact throughout the research journey [15,16].

Despite their significant contributions, the CRN role is often misunderstood or underappreciated. Several studies have reported that healthcare professionals and clinical staff may have an incomplete understanding of the CRN scope of practice, frequently associating the role primarily with protocol management, documentation, and data collection activities [17,18,19]. This misunderstanding creates significant barriers to their acceptance as a fundamental part of the multidisciplinary team. Furthermore, it risks overshadowing CRNs’ identity as a healthcare professional who actively engages in clinical care and advocates for participants throughout the research process.

The existing competency framework developed within the Clinical Research Nursing Domain of Practice comprehensively describes the expected scope of CRN responsibilities [10]. However, this framework primarily reflects normative or prescribed roles and does not necessarily capture how these activities are enacted in everyday patient-facing clinical research practice. This distinction between prescribed competencies and enacted practice is relevant because competency frameworks describe what CRNs are expected to do, whereas empirical studies provide insight into which activities are actually reported in practice and the level of involvement and autonomy associated with their performance.

Previous reviews have examined the broader competencies, qualifications, professional roles and responsibilities of CRNs, establishing that their practice encompasses clinical, research, coordination, educational, and advocacy functions. However, there remains limited evidence specifically synthesizing how patient-centered competencies are enacted in participant-facing clinical trial practice, which activities are most consistently reported in empirical studies, and how the degree of CRN involvement and autonomy varies across different healthcare, organizational, and regulatory settings. The present review addresses this gap by focusing specifically on enacted patient-centered activities rather than on the overall scope of CRN competencies and responsibilities.

Variation in enacted practice may be shaped not only by national context, but also by differences in organizational models, delegation of trial responsibilities, institutional policies, professional recognition, training requirements, and nursing autonomy within research teams. These contextual factors may influence whether patient-facing activities are performed independently by CRNs, shared with other professionals, or undertaken under physician supervision. Examining enacted patient-centered practice across empirical settings may therefore provide a complementary perspective on existing competency frameworks and broader reviews of the CRN role.

Therefore, this scoping review aims to examine how patient-centered activities are enacted by CRNs in clinical trials. By mapping the activities reported during interactions with research participants, the review provides an empirical perspective on how patient-centered CRN competencies are enacted across different clinical research contexts, complementing existing competency frameworks and broader descriptions of CRN roles and responsibilities.

## 2. Materials and Methods

### 2.1. Study Design

A scoping review was conducted following the methodological guidelines of the Joanna Briggs Institute (JBI) and was guided by the Preferred Reporting Items for Systematic Reviews and Meta-Analyses extension for Scoping Reviews (PRISMA-ScR) checklist (Supplementary File S1) [20,21]. The protocol for this scoping review was registered on the Open Science Framework (OSF; Registration ID: 3ge9k, URL: https://osf.io/3ge9k (accessed on 10 September 2026). This methodology was selected as the most appropriate approach for mapping the breadth, characteristics, and variability of evidence on patient-centered activities performed by CRNs, rather than evaluating intervention effectiveness or establishing causal relationships. The review aims to answer the following research question: “How are patient-centered activities enacted by Clinical Research Nurses in clinical trial practice across heterogeneous empirical and contextual settings?”

### 2.2. Data Source and Search Strategy

The literature search was performed in September 2024 and updated in April 2026 using the following databases: MEDLINE (via PubMed), CINAHL (via EBSCO), and EMBASE. A manual search of the reference lists of included studies was also performed to identify additional relevant articles not captured by the electronic search strategies. A gray literature search was performed using the following sources: GreyLit, OpenGrey and Google Scholar, as well as the websites of organizations dedicated to clinical research nursing. Gray-literature searches were conducted using predefined free-text terms related to clinical research nursing, CRN roles, competencies, skills, activities, and tasks.

The search strategy was developed with the support of an experienced medical librarian and was peer-reviewed by a senior researcher. A preliminary PubMed search was conducted to identify relevant free-text and controlled vocabulary terms based on the Population, Concept, and Context (PCC) framework (Supplementary File S2). The search strategy, including identified keywords and index terms, was then adapted for each database used for this scoping review. The complete search strategy strings are provided in Supplementary File S3.

### 2.3. Inclusion Criteria

Inclusion criteria were guided by the PCC framework. The population of interest included CRNs, defined in accordance with the ANA-IACRN framework [11], which characterizes CRNs as nurses primarily engaged in the direct care of participants enrolled in clinical trials. The conceptual focus was on patient-centered activities, defined as CRN activities involving direct interaction with research participants and supporting their clinical care, safety, understanding, engagement, decision-making, well-being, or continuity throughout trial participation, as described or self-reported by CRNs themselves. Technical activities were included only when directly related to participant care or safety. The context was limited exclusively to clinical trials, defined according to Good Clinical Practice (GCP) as studies conducted on human subjects to evaluate an investigational product [5]. Articles published in English within the last 20 years were eligible for inclusion. Studies conducted in decentralized clinical trial settings and those focused exclusively on pediatric trials were excluded because these contexts involve substantially different care, family involvement, and non-hospital-based settings that may affect the role and activities of CRNs. Consistent with JBI guidance for scoping reviews, critical appraisal of included studies was not performed, as the primary objective was to map and characterize the available evidence rather than to assess methodological quality.

### 2.4. Study Selection

Peer-reviewed articles from qualitative and quantitative studies, reviews, and self-reported experiences were imported into the Rayyan Intelligent Systematic Review System. Duplicate records were identified and removed; subsequently, six independent reviewers worked in pairs to perform study selection [22]. Each article was assessed in a double-blind manner by two independent reviewers, who first screened the titles and abstracts for eligibility according to the inclusion criteria and then assessed the full texts of articles deemed potentially eligible. At each stage of screening, conflicts were resolved through an assessment conducted by a third senior researcher.

### 2.5. Data Extraction

Four authors, in pairs, independently extracted data from each included article. Two other authors supervised the data extraction process. A Microsoft Excel spreadsheet was created specifically for the purpose of this study.

In studies including mixed professional populations, only CRN-specific data were included in the synthesis. The total participant count therefore reflects only CRNs whose data were reported separately. For secondary sources, only findings derived from primary studies that met the review inclusion criteria and were relevant to patient-centered CRN activities were retained. Data were synthesized descriptively. Reported activities meeting the operational definition of patient-centered practice were grouped into conceptually related domains through an iterative process conducted by the review team. The resulting domains were used to map patterns of enacted CRN practice across different clinical trial contexts. Findings were interpreted as reflecting the practices reported in the included studies rather than being representative of national practice overall. Methodological characteristics relevant to interpretation, including study design, sample characteristics, sampling approach, use of validated or study-developed instruments, and type of evidence contributed, were descriptively considered for each included source. These elements were used to support interpretation of the synthesis, without undertaking a formal methodological quality appraisal.

## 3. Results

**Figure 1:**
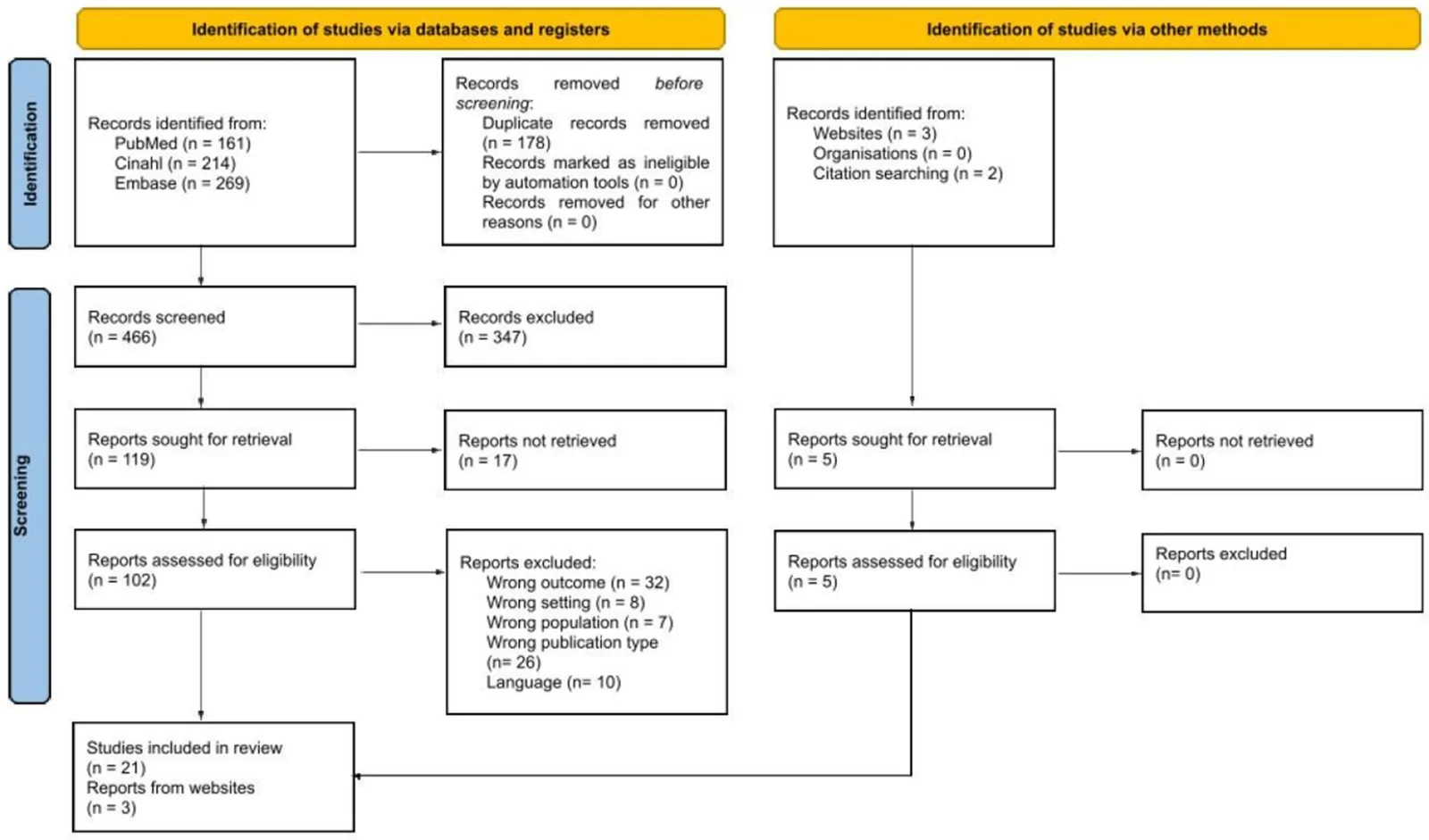
PRISMA flow diagram.

A total of 21 studies published between 2007 and 2024 were included in this scoping review. The study selection process is reported according to the PRISMA flow diagram [21] and is shown in Figure 1.

### 3.1. Characteristics of the Studies

Overall, 43% of the included studies were published in the past five years (*n* = 9). The majority were conducted in the United States [23,24,25,26,27,28,29] (*n* = 7, 33%), with Europe representing the second most common setting (*n* = 6, 29%) [30,31,32,33,34,35]. The remaining studies were carried out in China and South Korea (*n* = 4, 19%) [36,37,38,39], Australia and New Zealand (*n* = 3, 14%) [40,41,42], and South America (*n* = 1, 5%) [43]. Most of the included studies were cross-sectional (*n* = 12, 57%). Of these, three used the Clinical Trial Nursing Questionnaire (CTNQ), one used the Research and Clinical Trial Nurses Questionnaire (RCTNQ) and eight used online or web-based questionnaires or surveys. Five studies were literature reviews (24%), followed by qualitative studies (*n* = 2, 10%), one study describing the development of an instrument (*n* = 1, 5%), and one experience report (*n* = 1, 5%). The main characteristics of the included studies are presented in Table 1.

The included evidence was methodologically heterogeneous. In interpreting the synthesis, consideration was given to characteristics such as sample size and coverage, sampling approach, use of validated or study-developed instruments, and whether evidence was primary or secondary. Larger surveys using structured or validated instruments contributed broader self-reported descriptions of CRN activities, whereas qualitative and experience-based studies provided more contextualized accounts of role enactment. These methodological considerations are summarized in Supplementary File S4.

### 3.2. Population and Context

The 21 included studies represented heterogeneous international evidence involving CRNs and related clinical research professionals across multiple settings. Overall, more than 1800 participants contributed to the description of CRN activities, with the largest contributions deriving from studies conducted in China, the United States, and multicountry European surveys. Detailed characteristics of study populations are reported in Table 1.

### 3.3. Role of CRN: Patient-Centered Activities in Clinical Trials

Patient-centered activities performed by CRNs in clinical trials and reported across the included studies were grouped into eight domains: (1) screening and recruitment, (2) the informed consent process, (3) patient assessment and care, (4) investigational product management, (5) specimen management, (6) safety and adverse event monitoring, (7) education, advocacy, and support, and (8) study procedures and follow-up visit scheduling. Although presented as discrete categories for analytical clarity, several activities simultaneously reflected ethical, clinical, organizational, and communicative competencies, suggesting that patient-centered CRN practice is inherently multidimensional.

**Figure 2:**
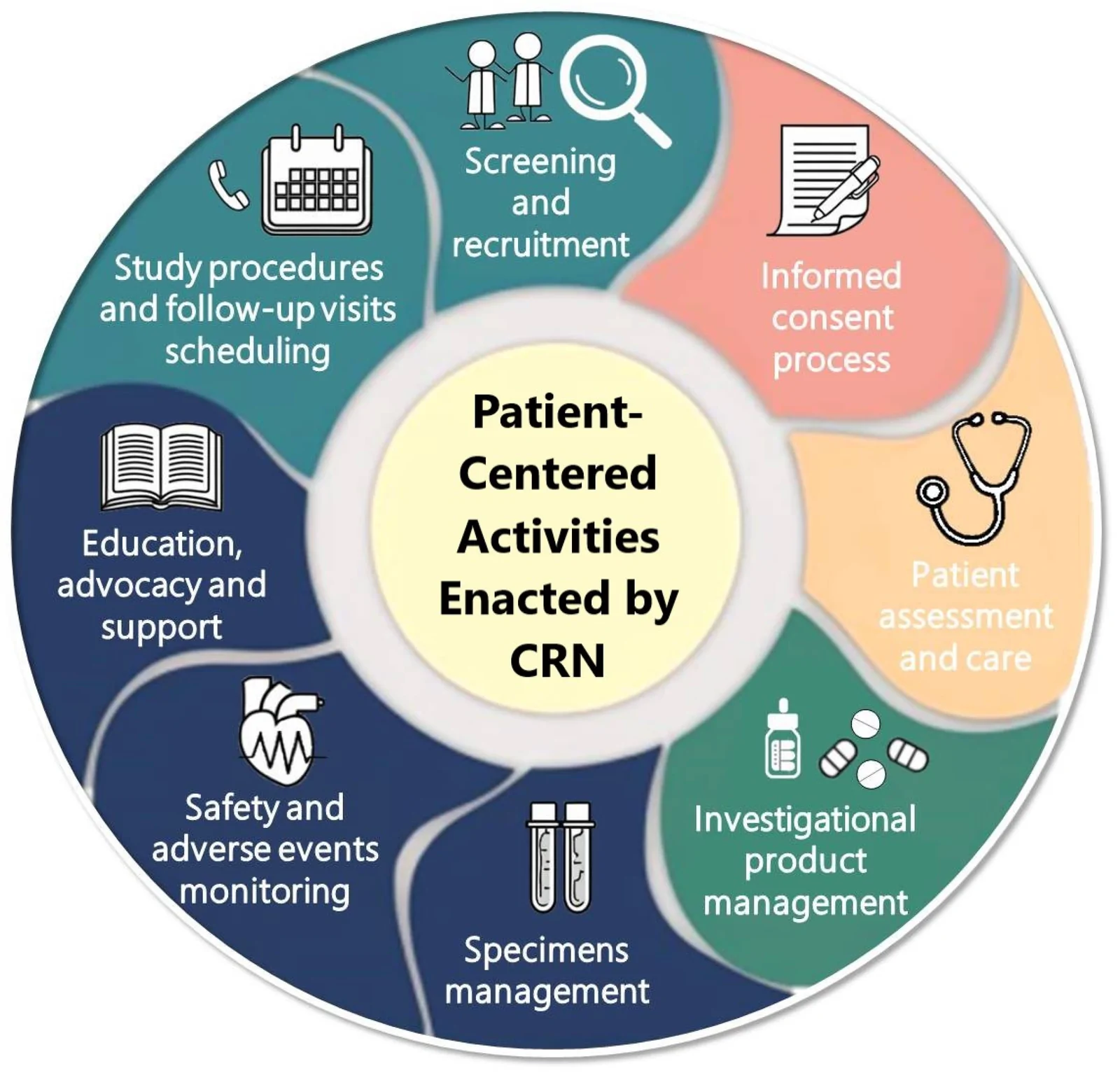
Patient-Centered Activities Enacted by Clinical Research Nurses in Clinical Trials. Conceptual representation of the patient-centered activity domains enacted by Clinical Research Nurses (CRNs) during clinical trials. These activities frequently overlap in practice and reflect multiple ethical, clinical, organizational, and communicative competencies.

**Figure 3:**
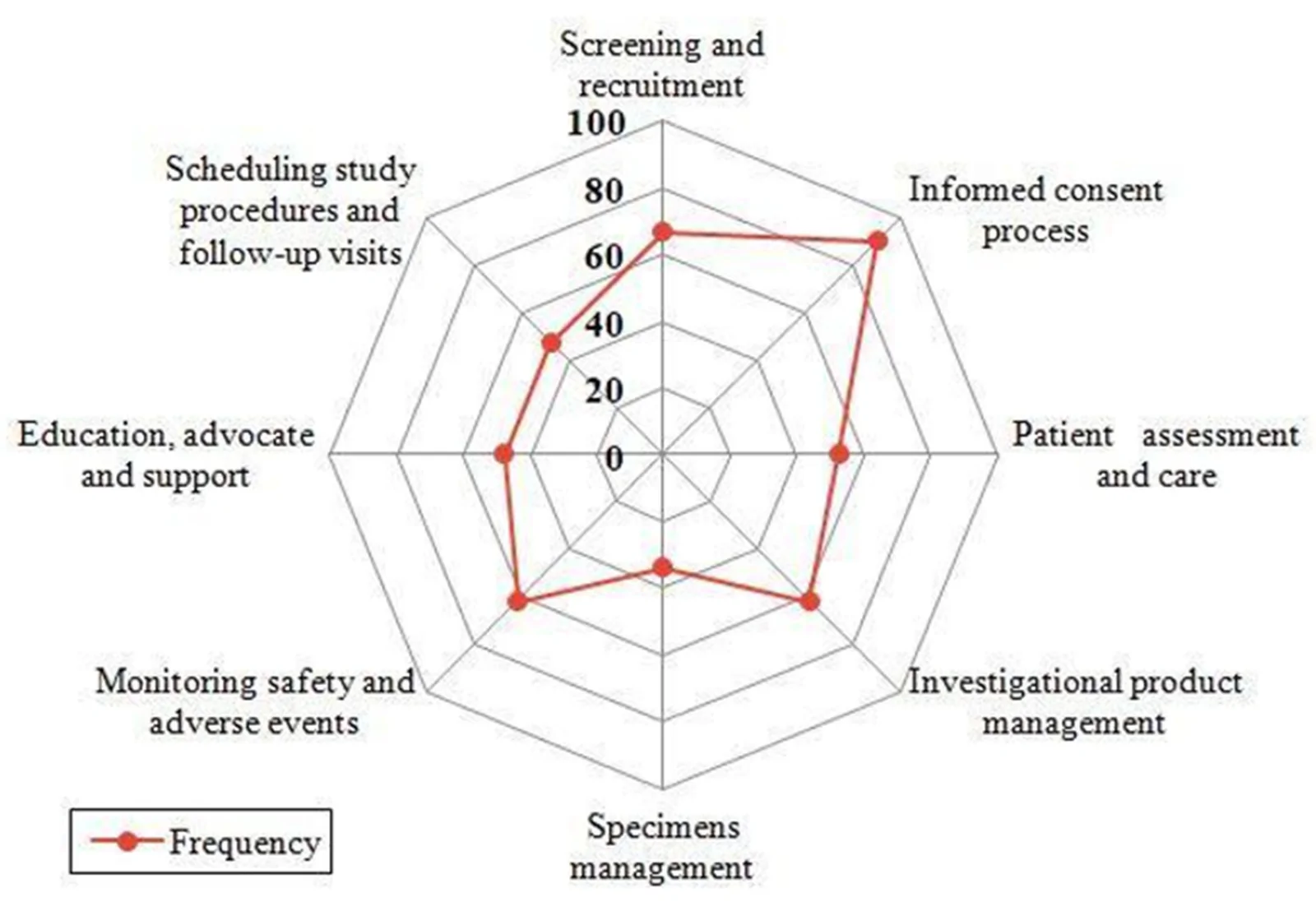
Proportion of included studies reporting each patient-centered activity domains in clinical research nursing practice across included studies. The radar plot shows the proportion of included studies in which each activity domain was reported. Percentages were calculated as the number of studies reporting a given domain divided by the total number of included studies (*n* = 21), multiplied by 100. These values indicate how frequently each domain was reported across the included studies.

Figure 2 provides a visual representation of these interconnected patient-centered activity domains. Table 2 reports patient-centered activities performed by CRNs across the included studies, and Figure 3 illustrates, using a radar plot, how frequently these activities were reported.

#### 3.3.1. Screening and Recruitment

CRNs are primarily responsible for, or collaborate with principal investigators (PIs) on, recruiting and enrolling patients in clinical trials, a task they frequently perform [26,36,38,40,41,42]. Participant recruitment involves ensuring regulatory compliance, conducting eligibility screening and enrollment procedures, maintaining enrolment logs, confirming participant eligibility, and developing recruitment strategies [25,31,33,41]. In contrast, tasks such as creating recruitment materials, including printed materials, websites, and study-related documents, are performed less commonly [33]. Screening and recruitment activities are described with varying levels of CRNs’ involvement across the included studies. In one study that involved CRNs from the US and the United Kingdom (UK), 86% of UK CRNs reported engaging in participant recruitment, with frequencies ranging from several times per month to multiple times per day. Conversely, 42% of US respondents indicated that recruitment was not part of their role [28]. Notably, Brinkman’s review described limited CRN involvement in recruitment activities in the Italian context [26]. In China, lower levels of theoretical knowledge about recruitment and retention strategies have been reported, although recruitment activities are still described as part of CRNs’ practice [37].

#### 3.3.2. Informed Consent Process

Patient enrollment in studies is contingent upon informed consent, which is obtained after participants have received appropriate information and their eligibility has been assessed. CRNs, through their strong patient advocacy skills, consistently provide information on the study procedures and potential risks; therefore, they are also actively involved in the informed consent process, including a verbal discussion about the study. As such, CRNs ensure patients’ and families’ comprehension and promote voluntary and informed decision-making [26,39]. Across the included studies, CRNs facilitate and support patients throughout the informed consent process in collaboration with the PI, although they are often not directly responsible for obtaining the patient’s signature on the consent form [28,30,31,32,33,35,36,37,38,41,42]. This supportive role is particularly important when addressing limited health literacy, which has been shown to positively influence enrolment outcomes [32,38]. In specific contexts, such as ICU studies in New Zealand involving unconscious patients, or in an experience report from Brazil involving CRNs working with minors or patients with mental disorders, CRNs consult with families to ascertain the patient’s wishes. In these cases, the CRN typically initiates the conversation with the family, which is subsequently continued by the physician [42,43]. A descriptive study conducted in Canada specifically explored CRNs’ views on the informed consent process. Using self-reported questionnaire data, Canadian CRNs described informed consent as an ethical responsibility and reported actively assessing participants’ ongoing willingness to continue participating in the study [23].

#### 3.3.3. Patient Assessment and Care

Across the included studies, the CRN’s role is characterized by a high level of involvement in patient care, applying evidence-based practices to plan and coordinate clinical nursing interventions [24,30,33,34,41]. One study reported that direct care accounted for a significant portion of CRNs’ responsibilities (>70%) [27]. Reported patient care activities included conducting comprehensive clinical assessments, performing protocol-specific procedures and tests (e.g., phlebotomy, electrocardiograms), implementing nursing interventions, and evaluating patient responses [26,32,33]. These activities were described across studies conducted in multiple contexts, including Australia, Canada, Italy, Japan, the UK, China and the USA [26,29,37]. In the management of trial-related patient care, CRNs were reported to require knowledge of the patient’s medical and personal history and hands-on clinical skills to deliver care, perform or coordinate physical or psychosocial assessments [39,40].

#### 3.3.4. Investigational Product Management

In cross-sectional studies based on online questionnaires involving CRNs from Sweden, the US, the UK, and Australia, investigational product management involved a range of tasks that are frequently performed and rated as highly important in these settings [28,33,41]. CRNs were responsible for ensuring compliance with protocols and verifying the dose to be administered [30,41]. Similarly, in studies conducted in New Zealand, CRNs also reported involvement in understanding investigational product risks, side effects, and their management [40,42]. In a multinational cross-sectional survey involving respondents from 37 European countries, the administration of investigational medicinal products emerged as one of the most frequently assigned tasks to CRNs, with reported involvement increasing during later phases of clinical trials [35]. Investigational product management included monitoring patient compliance and evaluating oral drug consumption [32]. In studies from China included in a scoping review by Xing et al., CRNs often collaborated with pharmacists throughout the comprehensive process of drug management [39]. A survey-based study involving CRCs from 10 hospitals in South Korea reported that investigational product management was rated as an important activity, although it was not always performed as frequently as necessary [38].

#### 3.3.5. Specimen Management

The management of biological specimens was frequently described as part of CRN practice, encompassing responsibilities for specimen collection, processing, storage, and transportation across various research settings. Studies from Sweden, Italy, South Korea, China, the USA, and Australia described specimen management as a commonly performed CRN activity in order to maintain research sample integrity and quality [24,30,33,36,38,39,41]. Reported responsibilities also included ordering supplies and ensuring compliance with research protocols for proper specimen collection and handling, a role observed across multiple nations [26]. For example, in pharmacokinetic studies, blood sample collection requires a higher number of test tubes compared to later-phase trials [31]. Furthermore, the experience of Brazilian CRNs has shown that storage at the appropriate temperature has been identified as a critical aspect of sample management [43].

#### 3.3.6. Safety and Adverse Events Monitoring

CRNs play a key role in monitoring safety and adverse event management in trials, as reported in the international comparison of CRN roles by Brinkman-Denney, which involved multiple countries, including Australia, Canada, India, Italy, Japan, New Zealand, South Korea, the UK, and the USA [26]. Survey studies reported that monitoring and reporting adverse events were among the most frequently performed activities by CRNs and were also rated among the most important [24,28,36,39,42]. The CRN is responsible for assessing participants’ health status and treatment response [38], with a crucial role in managing interventions, side effects, and adverse events [33]. This activity includes determining whether an event qualifies as an adverse event (AE) or a serious adverse event (SAE), considering any deviation or worsening of the participant’s baseline condition since enrollment [25]. The identification and reporting of adverse events according to protocol-specific criteria occur through physical assessments, subject-reported symptoms, clinical laboratory results, and diagnostic tests [25,30,43]. Moreover, the CRN is required to immediately report all AEs and SAEs to the principal investigator and the study sponsor [34,39].

#### 3.3.7. Education, Advocacy and Support

The CRN educational role is widely reported internationally [26,39] and it is recognized as a key resource for patients and their families in providing clarification on illnesses, treatments, and healthcare experiences [23,24,32,40]. CRNs assist participants in understanding study procedures, data collection, and research objectives, helping them make informed decisions about their participation. They provide explanations before, during, and after procedures and play a crucial role in alleviating anxiety and uncertainty stemming from participants’ lack of knowledge about clinical trials [30,39].

#### 3.3.8. Study Procedure and Follow-Up Visits Scheduling

CRNs play a crucial role in maintaining long-term follow-up, a key aspect of clinical trials [30,33]. A fundamental aspect of patient-centered care managed by CRNs in clinical trials is the scheduling of study procedures and follow-up visits. Ensuring appropriate follow-up is essential for monitoring adverse events, treatment outcomes, and overall patient well-being. In studies conducted in New Zealand and the USA, CRNs reported that they were directly responsible for the coordination and scheduling of follow-up visits, ensuring that participants receive continuous care throughout the study period [24,27,29,40,42]. Studies report that CRNs are central to organizing participant follow-up appointments [26] and maintaining contact with patients via phone to assess their progress and address any concerns [32].

### 3.4. Gray Literature

The gray literature reviewed included key sources that describe patient-centered activities performed by CRNs, which align closely with the findings of this review. Three sources provided direct or indirect testimonies on the role of CRNs [44,45,46]. Ness E., a nurse consultant at the Center for Cancer Research, highlights the core responsibilities of CRNs, emphasizing their involvement in the informed consent process, the scheduling of study procedures and follow-up visits, and their supportive role, which is crucial for adherence and participant retention [44]. Similarly, personal accounts by Gleason, a CRN for the UK’s NHS, provide an overview of CRN responsibilities. These include facilitating informed consent, recruiting patients, ensuring compliance with scheduled protocol visits, tissue and sample collection, and safety monitoring [45,46].

## 4. Discussion

This scoping review aimed to examine how patient-centered activities enacted by CRNs are reported in clinical trial practice across published empirical contexts. While many of the activities identified are reflected in existing competency frameworks, a key contribution of this review lies in examining how these activities are enacted and reported in empirical clinical trial settings. Rather than defining what CRNs should do, this review maps how patient-centered activities become visible in real-world settings, as reported by CRNs themselves, and how their enactment varies across studies and settings.

Although previous studies and competency frameworks have described the expected roles and responsibilities of CRNs, less attention has been paid to how these competencies are operationalized in everyday participant-facing practice. This gap is particularly significant given that clinical research nursing has been described as a relatively new and evolving discipline, in which clarity on professional roles, standards, and scope of practice is still developing across different national contexts [47]. The distinction between prescribed competencies and enacted practice is particularly relevant because organizational structures, regulatory environments, professional recognition, and the availability of formalized training pathways may substantially influence how CRNs interact with research participants and deliver patient-centered care [48].

Our analysis revealed that while these activities are commonly reported across studies conducted in different countries, their execution, frequency, and level of autonomy vary across settings. Across the included studies, CRNs were frequently described as involved in activities related to patient safety, ethical processes, and protocol-related care coordination. These variations indicate that patient-centered clinical research nursing is not uniformly enacted across settings and may be influenced by differences in organizational structures, professional roles, workforce models, and regulatory environments.

The distribution of reported activities across the included studies also suggests that not all domains of CRN practice are equally visible in the literature. Activities related to informed consent, recruitment, investigational product management, and safety monitoring were more frequently reported, whereas domains such as specimen management, patient assessment, and follow-up coordination appeared less consistently described. This uneven representation may reflect differences in role enactment across settings, but also possible differences in how CRN activities are documented and emphasized within empirical research. The predominance of activities related to recruitment, informed consent, investigational product management, and safety monitoring likely reflects their central importance for participant protection, ethical conduct, and trial integrity. Conversely, the lower visibility of other domains may indicate underreporting within the literature rather than reduced relevance in clinical research practice. A similar pattern has been observed in broader clinical research workforce studies, where the emphasis on protocol-driven tasks may overshadow the relational and coordinative dimensions of research nursing practice [49].

Over the past two decades, the CRN role has been described in the literature as ranging from a technical assistant to a clinically autonomous competent professional with specialized training in research ethics, regulatory frameworks, and patient engagement. This evolution is consistent with the broader trajectory of clinical research nursing as a specialty, which gained formal recognition in the United States through the IACRN and ANA in 2016, although equivalent recognition remains absent in many other countries [11,12,47]. The literature from the UK, USA, Canada, and Australia reports CRNs as directly involved in clinical activities such as informed consent, adverse event management, and investigational product administration. As reported by Xing et al. (2024) [39], the CRN is described not only as a task executor but as a coordinator and advocate for research participants, serving as a bridge between research teams and patients. Zardo et al. (2023) [50] emphasized that this duality, scientific and humanistic, requires CRNs to possess a wide range of clinical, organizational, and relational skills. Brioni and Magnaghi (2019) further argued that a person-centered care approach in clinical research enables CRNs to promote participants’ choices while simultaneously supporting study integrity, thereby humanizing the research process [51]. Collectively, these findings reinforce the view that CRNs operate at the interface between research and clinical care [52]. Unlike many research professionals whose responsibilities are primarily methodological or administrative, CRNs maintain direct therapeutic relationships with participants while simultaneously ensuring adherence to study protocols and regulatory requirements [53,54]. This dual function may represent one of the defining characteristics of contemporary clinical research nursing. However, this duality can also generate tension, as described by Tinkler et al. (2018), who identified a “caring-recruiting dichotomy” in which CRNs may experience conflict between their patient advocacy responsibilities and institutional expectations regarding recruitment targets [55].

This review reports variation in CRNs’ responsibilities across studies conducted in different healthcare and organizational contexts. While direct patient care, administration of investigational products, and monitoring of adverse events are responsibilities consistently reported across settings, including studies conducted in the UK, USA, several European countries, South Korea, and Australia, other activities show variability. For instance, in studies conducted in the UK, Australia, USA, and Canada, CRNs were reported to be involved in obtaining informed consent and conducting participant recruitment, although some US studies reported that these activities were not performed as part of the CRN role. In contrast, studies conducted in Italy, China or South Korea more frequently described these activities as physician-supported or supervised [26,30,36,38]. Kunhunny and Salmon (2017) described CRNs in the Indian context as assuming responsibilities as extensive as those of a principal investigator, whereas an Italian study described a more task-oriented role focused on specimen handling and patient monitoring [47].

Such variability may reflect differences in professional scope, regulatory requirements, educational pathways, institutional organization, and the delegation of responsibilities within research teams. These contextual factors may influence the level of autonomy with which CRNs perform patient-centered activities, although they were not systematically examined across the included studies. Similar patterns have been described in other advanced nursing specialties, where professional autonomy has been associated with healthcare system characteristics and national regulatory frameworks. A recent comparative review by Cao et al. (2025) further highlighted differences in role positioning, divisions of responsibilities, and career development pathways between developed and developing countries as factors that may influence clinical trial delivery [56].

Similarly, educational and advocacy functions, as well as the scheduling of study visits, are reported with varying levels of engagement across studies. In a study conducted in New Zealand and in an experience report involving two CRNs in Brazil, CRNs are actively involved in supporting families of unconscious patients or participants with reduced decision-making capacity, contexts that are described as requiring ethical sensitivity and communication skills. Studies conducted in New Zealand and the United States also reported CRNs independently coordinating follow-up visits and maintaining continuous contact with participants, promoting adherence and ensuring protocol fidelity throughout the CT [24,27,29,40,42].

This variability highlights differences in the scope, autonomy, and contextual application of CRN competencies across settings. As reported by Martucci et al. (2020) [57], a lack of institutional recognition and standardized training pathways was described as a factor affecting CRN professional development in some health systems. Previous studies have also identified institutional recognition and standardized training pathways as relevant to CRN professional development [58]. Spilsbury et al. (2008) similarly reported associations between CRN involvement at research sites and aspects of trial delivery, including communication, recruitment, and patient compliance with protocols [59].

A recurring theme across the literature is the ethical and relational dimension of the CRN role. CRNs are reported to be responsible for ensuring that participants are fully informed, willing, and supported throughout their involvement in a trial. Brown et al. (2019) and Hall et al. (2012) emphasized how the consent process, particularly in vulnerable populations, is ethically complex and emotionally demanding [60,61].

Godskesen et al. (2023) further explored these challenges in a qualitative study of Swedish CRNs, identifying threats to voluntariness including time constraints, information overload, and excessive reliance on physicians’ recommendations, while also describing proactive strategies CRNs employ to safeguard participant autonomy [15]. Larkin et al. (2019) reported that CRNs experience specific ethical challenges arising from the tension between protocol implementation and the duty of patient advocacy, including conflicted allegiances between the needs of participants, the desire to advance science, and the requirements of the research relationship [62].

Moreover, CRNs are often reported as the primary point of contact for participants. Their role in explaining procedures, managing anxiety, and advocating for patient rights is described in the literature as important for participant retention and trust. The literature highlights CRNs’ educational and supportive functions as essential in delivering ethical, humanized research care [58].

By focusing specifically on patient-centered activities, this review provides a focused synthesis of how CRN competencies are reported in participant-facing clinical trial practice. Although this review focused exclusively on patient-centered activities, deliberately excluding study-related and administrative tasks, this scope allowed for a focused analysis of CRN interactions with research participants. This review reports relational and clinical dimensions of CRNs’ practice that are less frequently detailed in broader role analyses. Although competency frameworks describe broad, multidimensional domains of CRN practice, the empirical literature in this review appears to emphasize patient-facing clinical and coordinative activities more prominently than other competency domains.

This scoping review presents several limitations that may affect the interpretation of its findings. First, although grey literature was partially included, dissertations and theses were excluded. This may have resulted in the omission of insights into local practices and undocumented experiential knowledge related to CRN activities across various settings.

Second, the review was limited to publications in English. Consequently, the literature from non-English-speaking contexts, particularly where CRNs operate under different terminologies or frameworks, may have been inadvertently excluded, introducing a potential language and terminology bias.

Third, contextual factors such as professional scope, regulatory frameworks, and institutional organization were not consistently reported across studies. Differences in study design and data collection methods may also have influenced which CRN activities were captured and reported, limiting a structured comparison of the factors underlying variation in CRN practice.

Finally, despite the international scope of this review, geographic representation was uneven, with a predominance of studies from the USA and Europe and limited evidence from regions such as Africa and the Middle East. This imbalance, together with the overall limited evidence base, may reduce the breadth of the international picture provided by the review.

These limitations suggest the need for future systematic reviews that incorporate rigorous quality assessment, broader linguistic inclusion, and greater geographic diversity. Furthermore, future reviews may consider integrating local and non-formalized knowledge sources to map the scope and variation in the CRN role.

Based on the evidence mapped in this review, several implications for CRN education and workforce development can be considered. The activities identified could inform the operationalization of patient-centered CRN competencies into measurable competency indicators and outcomes. For example, competency assessment could consider the ability to support informed consent and participant education, manage patient-facing aspects of investigational product administration, and coordinate follow-up. Potential indicators could include completion of competency-based training, assessment of specific activities, and the level of independence or supervision required for their performance.

National and international organizations may consider further defining and supporting the CRN role through certification pathways, education programs, and institutional policies to fully leverage its potential. Integrating ethical, relational, and technical competencies in CRN training may be relevant to the evolving complexity of clinical trials. Healthcare systems may consider formally recognizing CRNs as specialized professionals with defined career trajectories. Universities and academic institutions may consider incorporating research methodology and ethics into undergraduate and graduate nursing curricula.

Future research may prioritize outcome-based evaluations of CRN practices, focusing on measurable impacts such as participant recruitment and retention, patient satisfaction, protocol adherence, and data quality. Comparative studies between countries with different regulatory frameworks could further explore differences in CRN roles and practices. Additionally, qualitative research exploring CRNs’ lived experiences of patient-centered practice across diverse clinical trial settings would complement the predominantly survey-based evidence identified in this review.

## 5. Conclusions

This scoping review mapped how patient-centered activities performed by Clinical Research Nurses are described across different clinical trial contexts. The findings demonstrate that CRNs are involved in a broad range of relational, clinical, educational, and coordinative activities that support participants throughout the trial pathway, while also revealing variability in how these activities are enacted across studies and settings. By focusing specifically on patient-centered practice, this review provides a more comprehensive understanding of the clinical and relational dimensions of the CRN role as reported in the current literature.

## Figures and Tables

**Table 1:** Characteristics of included studies.

Author, Year, Country	Design and Methods	Population	Context	Concept: Research Participants-Centered Activities
Backman Lönn et al., 2022 [33], Sweden	Cross-sectional with the Clinical Trial Nursing Questionnaire (CTNQ). Rated frequency (0–4) of activities	161 CRNs	Primary care, hospitals and universities in Sweden	Subject recruitment (2.61 ± 0.76), Informed consent process (3.24 ± 0.59), Investigational product management (3.19 ± 1.08), Implementation and evaluation (2.87 ± 0.71)
Bell, 2009 [40], New Zealand	Literature review	37 papers	New Zealand research nursing environment	Screening and recruitment. Patient assessment. Study treatment. Monitoring patient’s response. Coordination of patient’s treatment and follow-up. Patient/family support and education. Facilitating informed consent.
Bevans et al., 2011 [24], USA	Cross-sectional with a web-based questionnaire	287 CRNs 74 RNCs 18 Nurses 33 Others	Biomedical research center in Maryland	Direct care (99%), Monitor for potential adverse events (94%), Teaching (92%), Participate in recruitment (12%), Scheduling study procedures (60%), Facilitate inquiries and concerns (79%), Facilitate initial and ongoing informed consent (50-70%), Supporting for participating in study (61%), Coordinate the collection of research specimens (93%)
Brinkman-Denney, 2013 [26], USA	Literature review	10 studies	North America, Europe and Asia-Pacific research clinical settings	Assessed participants. Monitor safety. Report issues or adverse experiences. Manage medications and specimens. Educate and advocate for participants. Participate in the informed consent process. Schedule visits. Collaborate on recruitment
C. Catania, 2012 [25], USA	Overview	903 literature results	/	Detection, gathering, assessment of AEs and SAEs
Cantini, 2007 [23], Canada	Cross-sectional with a questionnaire	65 CRNs	Clinical trial research team from University-affiliated hospitals in Quebec	Being involved in the informed consent process before, during, and after securing (75%)
De Aguiar, 2010 [43], Brazil	Experience report	2 CRNs	Randomized, blinded, phase III clinical trial	Clarify doubts, participate in collection of biological material, obtain Free and Informed Consent, be responsible for administering, accounting, returning the research product, and registering clinical problems and adverse events
Fisher et al., 2022 [28], USA	Cross-sectional with an online questionnaire	103 CRNs 130 RCNs or Others	Academic medical and clinical research center for in/outpatient in USA and UK	Recruitment (68% USA/ 86% UK), Monitoring for an adverse event (78% USA/ 88% UK), Administration of an investigational research product (83% USA/ 98% UK), Obtaining informed consent (62% USA/ 94% UK)
G. Catania et al., 2012 [30], Italy	Cross-sectional with CTNQ. Rated frequency (0–4) of activities	30 CTNs	Oncology hospitals in Italy	Subject Recruitment (1.77), Informed Consent Process (2.29), Investigational product management (2.66), Implementation and evaluation (2.51)
Hao et al., 2022 [37], China	Cross-sectional with an online questionnaire. Rated knowledge and performance in practice (0–5)	638 CRNs	Clinical sites, hospital departments, and clinical trial offices in China	Gaps between knowledge and practice: Patients’ Management (1.71), Informed consent (1.57), Participants’ recruitment and retention (−0.69)
Jeong, 2007 [36], South Korea	Cross-sectional with a questionnaire	71 investigators 79 CRNs	77 research institutions in South Korea	Screening subjects (82%), Recruiting subjects (52%), obtaining informed consent (79%), monitoring and reporting adverse events (80%), collecting specimens (76%)
Jones et al., 2022 [29], USA	Qualitative study with an internet-based four open-ended questionnaire and qualitative content analysis	30 CRNs 19 Coordinator 14 CRN coordinators 27 Other	Academic medical centers, biopharmaceutical companies, clinical government agencies and CRO	Nursing care, Relationships with participants and families, Ensuring continuity of care during the study treatment and in long-term follow-up, Teaching
MacArthur, 2014 [31], UK	Cross-sectional with an online survey	108 CRNs	NHS and universities in Scotland	Recruitment (94%), Screening potential participants (91%), Being involved in the informed consent process (82%)
Mackle, 2019 [42], New Zealand	Qualitative descriptive study using semi-structured interviews analyzed by manual content and thematic techniques	11 research nurses 6 principal investigators 6 nurse managers	ICUs in New Zealand	Identification of potential patients (11/11), Participates in consent process (9/11), Administration or facilitation of study treatment (10/11), Patient follow-up (10/11), Notices serious adverse events (8/11)
Milani et al., 2017 [32], Italy	Instrument development study	7 CTNs	Oncology research hospital in Italy	First visit for study proposal, Informed consent signing, Patient assessment, Drug reconciliation and administration, Education, Blood collection, Electrocardiogram performing, Phone contact with patient
Park, 2022 [38], South Korea	Cross-sectional with CTNQ. Rated frequency (0–4) of activities	102 CRCs	Clinical trial centers at tertiary hospitals and CRO in South Korea	Subject recruitment (2.81 ± 0.73), Informed consent process (3.22 ± 0.70), Investigational product management (2.83 ± 0.87), Implementation and evaluation (3.04 ± 0.72)
Peralta, 2024 [34], Spain	Review	/	/	Providing and coordinating clinical care, assuring ongoing maintenance of informed consent, education, monitoring patient safety, assessing and advocating for participants, administering investigational medicinal products
Purdom et al., 2017 [27], USA	Cross-sectional with a web-based questionnaire	167 oncology nurses	Oncology clinical trial	Monitor adverse events, Teach and explain to participants/family, Care, Help with inquiries and concerns, Schedule study procedures, Support with reasons and goals, Recruit participants, Facilitate initial and ongoing informed consent
Salmanton-García et al., 2024 [35], Germany	Cross-sectional with an online questionnaire	37 European countries	European organizations, societies and institutions from the VACCELERATE Network	Investigational medicinal products administration (81.1% of countries in phase I trials, 86.5% in II and III, 89.2% IV), Providing patient training for new medicines or procedures (>70% for all trials), Taking patient informed consent (45.9% I and II trials, 51.4% III and IV)
Wilkes et al., 2012 [41], Australia	Cross-sectional with a Research and Clinical Trial Nurses Questionnaire (RCTNQ). Rated frequency (0–4) of activities	67 CTNs	Health services and universities in Australia	Subject recruitment (3.07 ± 0.79), Informed consent process (3.16 ± 0.82), Implementation and evaluation (3.11 ± 0.85), Investigational product (2.69 ± 1.15)
Xing et al., 2024 [39], China	Scoping review	26 articles	/	Specimen management, Drug management, Subject management, Direct care and subject protection (support, communication, obtaining informed consent, monitoring and identifying adverse events), Training and education

Legend: CRN—Clinical Research Nurse; CRO—Contract Research Organization; CTN—Clinical Trial Nurse; CT—Clinical Trial; RCTNQ—Research and Clinical Trial Nurses Questionnaire; RNC—Research Nurse Coordinator; UK—United Kingdom; USA—United States of America.

**Table 2:** Overview of patient-centered activities performed by Clinical Research Nurses across included studies.

Author, Year, Country	Screening and Recruitment	Informed Consent Process	Patient Assessment and Care	Investigational Product Management	Specimens Management	Safety and Adverse Events Monitoring	Education, Advocacy and Support	Study Procedures and Follow-Up Visits Scheduling
Backman Lönn et al., 2022 [33], Sweden	x	x		x		x		x
Bell, 2009 [40], New Zealand	x	x	x	x		x	x	x
Bevans et al., 2011 [24], USA	x	x	x		x	x	x	x
Brinkman-Denney, 2013 [26], USA	x	x	x	x	x	x	x	x
C. Catania, 2012 [25], USA						x		
Cantini, 2007 [23], Canada		x					x	
De Aguiar, 2010 [43], Brazil		x		x	x	x	x	
Fisher et al., 2022 [28], USA	x	x		x		x		
G. Catania et al. [30], 2012, Italy	x	x	x	x		x		x
Hao et al., 2022 [37], China	x	x	x					
Jeong, 2007 [36], South Korea	x	x			x	x		
Jones et al., 2022 [29], USA			x				x	x
MacArthur, 2014 [31], UK	x	x						
Mackle, 2019 [42], New Zealand	x	x		x		x		x
Milani et al., 2017 [32], Italy	x	x	x	x	x		x	x
Park, 2022 [38], South Korea	x	x		x				x
Peralta, 2024 [34], Spain		x	x	x	x	x	x	
Purdom et al., 2017 [27], USA	x	x	x			x	x	x
Salmanton-García et al., 2024 [35], Germany		x		x				
Wilkes et al., 2012 [41], Australia	x	x	x	x				
Xing et al., 2024 [39], China		x	x	x	x	x	x	

Legend: This table illustrates the presence of specific patient-centered activities performed by CRNs, as reported in the included studies. An “x” indicates that the activity was reported in the corresponding study.

## Data Availability

No new data were created or analyzed in this study. Data sharing is not applicable to this article.

## Supplementary Materials

The following supporting information can be downloaded at: https://www.mdpi.com/article/10.3390/healthcare14183111/s1/, Supplementary File S1_PRISMA-ScR-Checklist; Supplementary File S2_Framework Participants, Concept and Context; Supplementary File S3_Search Strategy; Supplementary File S4_Methodological characteristics.

## Abbreviations

The following abbreviations are used in this manuscript:

AE	Adverse Event
ANA	American Nurses Association
IACRN	International Association of Clinical Research Nurses
CRN	Clinical Research Nurse
CRO	Contract Research Organization
CTN	Clinical Trial Nurse
CT	Clinical Trial
JBI	Joanna Briggs Institute
PCC	Population, Concept, and Context
PI	Principal Investigator
PRISMA-ScR	Preferred Reporting Items for Systematic Reviews and Meta-Analyses Extension for Scoping Reviews
RCTNQ	Research and Clinical Trial Nurses Questionnaire
RNC	Research Nurse Coordinator
SAE	Serious Adverse Event
UK	United Kingdom
US	United States of America
