# Supplementary material for: Patient-Centered Activities Performed by Clinical Research Nurses in Clinical Trials: A Scoping Review of Enacted Practice
Source: Healthcare (Basel). 2026 Sep 20;14(18):3111. doi: 10.3390/healthcare14183111 (PMC13606372; doi:10.3390/healthcare14183111)
Supplement: Supplementary file 1 [file healthcare-14-03111-s001.zip › Supplementary File S2_Framework Participants, Concept and Context.pdf]

Supplementary File S2_Framework Participants, Concept and Context

**Participants:**	**Concept:**	**Context:**
Clinical research nurse	Role	Clinical trial
Research nurse	Activities	Controlled
Study nurse	Tasks	Clinical Trial
Clinical trial nurse	Competences	Randomized
Clinical research nursing	Skills	Controlled Trial
Clinical Nursing Research	Assignments	RCT
CRN	Responsibility	CT
CTN		

Legend: The participants included nurses involved in clinical research activities, regardless of the specific title used. These terminological differences were deliberately recognized to ensure comprehensive representation of all relevant professional roles. CRN=Clinical research nurse; CTN=Clinical trial nurse; RCT=Randomized Controlled Trial; CT= Clinical trial
