# Supplementary material for: Patient-Centered Activities Performed by Clinical Research Nurses in Clinical Trials: A Scoping Review of Enacted Practice
Source: Healthcare (Basel). 2026 Sep 20;14(18):3111. doi: 10.3390/healthcare14183111 (PMC13606372; doi:10.3390/healthcare14183111)
Supplement: Supplementary file 1 [file healthcare-14-03111-s001.zip › Supplementary File S3_Search Strategy.pdf]

Supplementary File S3_Search Strategy

Database	Framework	Terms
PubMed	**P**	(("Clinical Nursing Research"[MeSH Terms] OR "Clinical Nursing Research" [Title/Abstract] OR "clinical research nurse*"[Title/Abstract] OR "research nurse*"[Title/Abstract] OR "study nurse*"[Title/Abstract] OR "clinical trial nurse*"[Title/Abstract] OR "research nurse coordinator*"[Title/Abstract]) AND
	**C**	("nurse s role"[MeSH Major Topic] OR "Competenc*"[Title/Abstract] OR "clinical competence"[Title/Abstract] OR "Skills"[Title/Abstract] OR "Activities"[Title/Abstract] OR "Tasks"[Title/Abstract])) AND
	**C**	("clinical trials as topic"[MeSH Terms] OR ("trial*"[Title/Abstract] OR "randomized"[Title/Abstract] OR "quasi-randomized"[Title/Abstract]) OR "clinical trial"[Title/Abstract] OR "randomized controlled trial"[Title/Abstract])
Cinahl	**P**	((MH "Clinical Nursing Research") OR (MH "Clinical Research Nurses") OR (MH "Research Nurses") OR TI "clinical nursing research" OR AB "clinical nursing research" OR TI "research nurse coordinator*" OR AB "research nurse coordinator*" OR TI "study nurse*" OR AB "study nurse*" OR TI "research nurse*" OR AB "research nurse*" OR TI "clinical research nurse*" OR AB "clinical research nurse*") AND
	**C**	((MH "Nursing Role") OR (MH "Clinical Competence") OR (MH "Nursing Skills") OR (MH "Task Performance and Analysis") OR "nurse s role" OR "Competenc*" OR "Task*" OR "clinical competence" OR "skill*" OR "activities") AND
	**C**	((MH "Clinical Trials") OR (MH "Randomized Controlled Trials") OR "clinical trial*" OR "quasi-randomized" "randomized trial*")

Embase	**P**	('clinical nursing research':ab,ti OR 'clinical nursing research'/exp OR 'clinical research nurse*':ab,ti OR 'research nurse*':ab,ti OR 'study nurse*':ab,ti OR 'clinical trial nurse*':ab,ti OR 'research nurse coordinator*':ab,ti) AND
	**C**	('competence'/exp OR 'competence':ab,ti OR 'nurse s role':ab,ti OR 'competencies':ab,ti OR 'competency':ab,ti OR 'clinical competence':ab,ti OR 'skills':ab,ti OR 'activities':ab,ti OR 'tasks':ab,ti) AND
	**C**	('clinical trial'/exp OR 'clinical drug trial':ab,ti OR 'major clinical trial':ab,ti OR 'trial, clinical':ab,ti OR 'clinical trial*':ab,ti OR 'trial*':ab,ti OR 'randomized controlled trial':ab,ti)
GreyLit, OpenGrey, Google Scholar, Clinical Research Nursing organisations/associations		clinical research nurse; clinical research nursing; research nurse; study nurse; clinical trial nurse; role; competencies; skills; activities; tasks
