# Supplementary material for: Patient-Centered Activities Performed by Clinical Research Nurses in Clinical Trials: A Scoping Review of Enacted Practice
Source: Healthcare (Basel). 2026 Sep 20;14(18):3111. doi: 10.3390/healthcare14183111 (PMC13606372; doi:10.3390/healthcare14183111)
Supplement: Supplementary file 1 [file healthcare-14-03111-s001.zip › Supplementary File S4_Methodological characteristics.pdf]

Supplementary File S4_ Methodological characteristics relevant to interpretation

Study	Methodological contribution and interpretive considerations
Backman Lönn et al., 2022	Uses the validated Swedish CTNQ, providing standardized self-reported activity-frequency data in a relatively large CRN sample. Strategic sampling from different regions and settings in Sweden, although no complete national CRN register was available.
Bell, 2009	Secondary synthesis of 37 papers on research nursing, interpreted within the New Zealand nursing context; not based on primary CRN-level data.
Bevans et al., 2011	Large web-based survey using a validated Clinical Research Nurse Role Delineation Measure, providing standardized self-reported activity-frequency data; conducted at a single research center.
Brinkman-Denney, 2013	Secondary synthesis of 10 selected articles comparing collaborative CRN roles across countries; not based on primary CRN-level data.
C. Catania, 2012	Narrative overview focused on CTN safety reporting; not based on primary CRN-level data.
Cantini, 2007	Multicenter survey using a study-developed questionnaire with pilot testing for face validity and reliability; convenience/network sampling of hospital-based CTNs.
De Aguiar, 2010	Experience report from two nurses involved in a phase III randomized trial; provides detailed practice-based insight but reflects a very limited experiential sample.
Fisher et al., 2022	Survey using using the Clinical Research Nurse Role Delineation Measure developed by Bevans et al., providing standardized self-reported activity-frequency data; comparisons were limited to USA and UK respondents.
G. Catania et al., 2012	Nationwide Italian oncology survey using the validated Italian CTNQ, providing standardized self-reported activity-frequency data.
Hao et al., 2022	Large self-report survey from different employment settings and city tiers in China; based on established ONS/IACRN competency frameworks; The instrument was study-developed from these frameworks but not reported as formally validated.
Jeong et al., 2007	Survey using a study-developed self-report questionnaire with pilot-tested content validity; CRNs were recruited across South Korea through multiple convenience and network-based sources.
Jones et al., 2022	Qualitative content analysis of open-ended responses from experienced CRNs; provides contextual depth rather than activity-frequency estimates.

MacArthur et al., 2014	Broad Scottish CRN survey using a study-developed, non-externally validated questionnaire; self-selected respondents from multiple clinical research settings.
Mackle, 2019	Qualitative study across all six eligible Level III ICUs in New Zealand, using stakeholder triangulation and multiple strategies to support analytic rigor.
Milani et al., 2017	Instrument-development study integrating literature and the experience of seven CTNs from one oncology center, with direct observation of activities and retrospective testing across 141 trials.
Park et al., 2022	Survey using the validated Korean CTNQ, providing standardized self-reported activity-frequency data across 10 hospitals in South Korea.
Peralta, 2024	Broad narrative review of clinical trial professional roles and challenges; not based on primary CRN-level data.
Purdom et al., 2017	Survey in a convenience CRN oncology sample using the Clinical Research Nurse Role Delineation Measure developed by Bevans et al.; self-reported activity-frequency data were analyzed using principal components analysis to identify dimensions of practice specific to oncology.
Salmanton-García et al., 2024	Multinational survey across 37 European countries, providing percentage-based data on research nurse tasks assigned across different trial types and phases; 74% country-level response rate.
Wilkes et al., 2012	National Australian survey using an adapted RCTNQ based on the validated CTNQ, providing standardized self-reported activity-frequency data; recruitment relied on indirect network-based sampling because no national CTN register was available.
Xing et al., 2024	Scoping review of 26 studies using both English- and Chinese-language databases; provides broad secondary synthesis of CRN characteristics and competencies rather than primary activity-frequency data.

Legend: The comments summarize methodological features relevant to the interpretation of each source, including sampling, use of validated or study-developed instruments, type of evidence, and scope of reporting. CRN=Clinical research nurse; CTN=Clinical trial nurse; CTNQ=Clinical Trial Nurses Questionnaire; UK=United Kingdom; USA= United States of America
